# Contexts Control Negative Contrast and Restrict the Expression of Flavor Preference Conditioning

**DOI:** 10.1037/xan0000091

**Published:** 2016-01

**Authors:** Joseph M. Austen, David J. Sanderson

**Affiliations:** 1 Department of Psychology, Durham University

**Keywords:** learning, memory, conditioning, feeding, mice

## Abstract

Consumption of a high concentration of sucrose can have either a detrimental, negative contrast effect or a facilitatory, preference conditioning effect on subsequent consumption of a low concentration of sucrose, depending on the cues that are present during consumption. The role of context and flavor cues in determining these effects were studied using analysis of the microstructure of licking in mice. Exposure to a high concentration followed by exposure to a low concentration resulted in a transient reduction in mean lick cluster size, which was context dependent (Experiment 1). However, there was no change in the total number of licks or overall consumption. When a flavor that had previously been paired with a high concentration was paired with a low concentration, there was an increase in the total number of licks, and overall consumption, but no change in the mean lick cluster size (Experiment 2). Pairing a high concentration with a flavor in a particular context before pairing the context and flavor compound with a low concentration resulted in abolishing the expression of the flavor preference conditioning effect on the total number of licks and consumption (Experiment 3). These results demonstrate that although context and flavor cues have dissociable effects on licking behavior, their interaction has an antagonistic effect on the behavioral expression of memory.

Cues that are present during consumption can come to control behavior and determine how food is consumed and the extent to which it is consumed. However, whereas some cues increase feeding, other cues reduce feeding. The purpose of the current study was to assess whether these facilitatory or detrimental effects on feeding reflect independent or interacting processes.

It is well established that initial exposure to sucrose can have a number of effects on subsequent consumption of sucrose. In flavor preference conditioning, pairing a flavor with a high sucrose concentration (conditional stimulus [CS]+) and another flavor with a low sucrose concentration (CS–) leads to subsequent greater consumption of the CS+ than CS– when both are paired with a low sucrose concentration (e.g., Harris & Thein, 2005). In the successive negative contrast procedure, exposure to a high sucrose concentration (in the absence of any flavors) leads to a subsequent reduction in consumption of a low sucrose concentration compared with a condition in which animals only receive exposure to the low concentration (e.g., Flaherty, Becker, & Pohorecky, 1985). The successive negative contrast effect has been found to be dependent on context cues (Daniel, Wood, Pellegrini, Norris, & Papini, 2008).

Although both successive negative contrast and flavor preference conditioning are reliably demonstrated phenomena, it is not clear what circumstances allow one effect to occur and not the other. Specifically, given that flavors are always consumed in the presence of context cues, it is not known whether flavor and context cues interact to determine behavior. It is possible that preference conditioning and negative contrast rely on independent processes, and a particular factor determines whether one or the other process is engaged during acquisition and/or expression of learning. Alternatively, there may be some commonality or interaction between the processes that determine preference conditioning and negative contrast, such that the process that determines negative contrast may potentially act against the process that determines preference conditioning. Consequently, performance of preference conditioning may be tempered by negative contrast, and vice versa. In favor of this latter argument, there is evidence that some failures to find flavor preference conditioning are a result of anticipatory negative contrast effects in which consumption of a solution is reduced if reliably followed by a high concentration of sucrose (E. Capaldi, Sheffer, & Pulley, 1989; Lucas & Timberlake, 1992).

The opposite effects of successive negative contrast and preference conditioning may be because of the nature of context and flavor cues. Thus, contexts and flavors may simply engage in different learning processes. This may be related to the different sensory properties of context and flavors, or may be because of the differences in the temporal nature of the experience of the cues, with flavors having a greater temporal correlation with sucrose than the ambient context cues that form an animal’s environment. In contrast, successive negative contrast and preference conditioning may depend on specific training and testing parameters. Procedures that have produced successive negative contrast have typically used substantial exposure to the high concentration prior to the shift to the low concentration (Flaherty, 1996). In contrast, flavor preference conditioning can be readily obtained within a couple of exposures to the flavor–sucrose pairings (Ackroff, Dym, Yiin, & Sclafani, 2009). The first two experiments of the present study examined this latter possibility by testing successive negative contrast and flavor preference conditioning under similar conditions. Specifically, Experiment 1 tested the role of context cues in producing successive negative contrast in mice using a within-subjects design. Experiment 2 used a similar design, but the role of context cues was substituted with flavor cues (see Table 1).[Table-anchor tbl1]

Experiment 3 assessed whether context and flavor cues interact in determining the expression of preference conditioning and successive negative contrast. This was achieved by confounding presentation of context and flavor cues during exposure and at test (see Table 1).

A secondary purpose of the study was to assess the conditions for producing successive negative contrast and flavor preference conditioning in mice using a microstructure analysis of licking behavior (Davis & Smith, 1992; Spector, Klumpp, & Kaplan, 1998). The use of mice in the behavioral assessment of learning has greatly increased over recent years, given the development of genetic manipulations. Therefore, it will be beneficial to increase the behavioral procedures that can be used for assessment of psychological processes in mice. So far, there have been few studies of negative contrast in mice, and, to our knowledge, those studies have only measured consumption and not the pattern of licking behavior elicited by a solution (e.g., Mustaca, Bentosela, & Papini, 2000). Similarly, to our knowledge, flavor preference conditioning in mice has not previously been studied using a microstructure of licking analysis. Importantly, we have found, in mice, that, similar to rats (Davis & Smith, 1992; Spector et al., 1998), mean lick cluster size (i.e., the number of licks made in quick succession before a pause) increases monotonically as a function of sucrose concentration, and that total licks follows an inverted U-shaped function, with licking (and overall levels of consumption) being greatest for intermediate sucrose concentrations (Austen & Sanderson, 2015). Thus, mean lick cluster size provides a potential measure of palatability that is independent of consumption (for a review, see Dwyer, 2012). In addition to an effect on overall levels of consumption, changes in mean lick cluster size have also been found in successive negative contrast (Grigson, Spector, & Norgren, 1993) and flavor preference conditioning (Dwyer, Pincham, Thein, & Harris, 2009) in rats. Therefore, in the present experiments, mean lick cluster size was assessed in addition to the total number of licks and overall levels of consumption.

## Experiment 1

The purpose of Experiment 1 was to test the role of context cues in successive negative contrast using a within-subjects design that is comparable with the within-subjects procedure typically used for flavor preference conditioning (e.g., Dwyer et al., 2009; see Table 1). A previous study in our laboratory examined successive negative contrast in mice using a between-subjects design in which one group of mice received exposure to 32% sucrose and then were shifted to 4% sucrose at test. Another group received 4% sucrose during both exposure and test. It was found that prior exposure to 32% sucrose resulted in a transient reduction in the size of lick clusters during exposure to a 4% solution at test (Austen & Sanderson, 2015). A possible account of that effect is that context cues retrieved a representation of the 32% solution at test, which resulted in a perceived reduction in the palatability of the 4% solution.

There is some evidence that context cues do determine successive negative contrast (Daniel et al., 2008). However, other studies have failed to detect a role of contextual cues (Flaherty, Hrabinski, & Grigson, 1990). In the study by Flaherty et al. (1990), a between-subjects design was used, in which one group received the exposure and test phases in the same context and another group received the phases in separate contexts. It is possible that such between-subjects designs lack the sensitivity to detect context-dependent effects. Therefore, in the current experiment mice were tested using a within-subjects design (see Table 1) similar to that used by Daniel et al. (2008). Mice received exposure to the 4% solution in Context A and exposure to a 32% solution in Context B. At test, mice were presented with the 4% solution in both Contexts A and B. If the successive negative contrast effect of reduced lick cluster size is determined by context cues, then mice will show smaller lick clusters in Context B than in Context A during the test.

Previous tests of the role of context cues in successive negative contrast have suggested that animals need a certain period of time to form a representation of the environment before consumption commences in order for the context to control licking (Daniel et al., 2008). Therefore, in the current experiment, the sipper tubes were available for only the last 10 min of each 15-min session so that mice were exposed to the context cues prior to the start of consumption.

### Method

#### Subjects

Eight female C57BL/6J/Ola mice (Charles River UK Ltd., Margate, United Kingdom) were used. Mice were housed in groups of four in a temperature-controlled room with a 12-hr light–dark cycle (lights on at 7:00 a.m.). Mice were approximately five months old at the beginning of the experiment and weighed between 20.1 and 22.7 g (*M* = 21.2 g). The mice had previously been used in an unrelated appetitive, magazine approach, conditioning experiment, conducted in a different room in operant boxes that were distinct from those used in the current experiment. Mice remained on a restricted diet for the duration of testing to maintain their weights at 85% of their initial free-feeding weights. Mice had ad libitum access to water in their home cages throughout the experiment. Testing always occurred when lights were on in the housing room.

#### Apparatus

Four identical operant chambers (interior dimensions: 21.6 × 17.8 × 12.7 cm; ENV-307W, Med Associates, St. Albans, VT), enclosed in sound-attenuating cubicles (ENV-022V, Med Associates) were used. The operant chambers were controlled by Med-PC IV software (Med Associates). The side walls were made from aluminum, and the front and back walls and the ceiling were made from clear Perspex. The chamber floors each comprised a grid of 24 stainless steel rods (0.32 cm diameter), spaced 0.79 cm apart and running perpendicular to the front of the chamber (ENV-307W-GFW, Med Associates). Retractable sippers (ENV-352AW, Med Associates) and a small hole in one wall of each chamber allowed sipper tubes to be extended into, and retracted from, the chambers. The sipper tubes (0.1 ml) allowed measurement of consumption by comparing the volume before and after testing. Contact lickometer controllers (ENV-250, Med Associates) allowed contacts between the mice and the sipper tubes to be recorded at a resolution of 0.01 s. A fan (ENV-025F, Med Associates) was located within each of the sound-attenuating cubicles and was turned on during sessions. A house light (28V, 100mA; ENV-315M, Med Associates) situated in the top center of the wall opposite the retractable sipper, and a clicker (2Hz, 75dB; ENV-335M, Med Associates) located to the left of the house light, were used to create distinct contexts. One context was created by the absence of sound and light (i.e., the house light and clicker were not presented), whereas the other was created by the presentation of the house light and clicker. Sucrose solutions used were 4% and 32% (wt/vol) commercially available sucrose dissolved in water.

#### Procedure

Mice received eight training sessions (one per day), consisting of two trials per session (with an intertrial interval of approximately 10 min). On one trial per session, mice were allowed to drink 4% sucrose in Context A, and on the other trial, they could drink the 32% sucrose in Context B. Each trial, the start of which was defined by placing the mouse in the chamber, lasted 15 min. For the first 5 min of each trial, the sipper tube was not extended into the chamber, thus preventing consumption of the solution. The sipper tube was only extended into the operant chamber for the final 10 min of the trial. For half of the mice, Context A was the quiet, dark context (i.e., the house light and clicker were not presented), and Context B was the context created by the presentation of the house light and clicker. For the remaining mice, the contingencies were reversed. Within these two groups, half of the mice followed a repeating ABBA context order across successive sessions, whereas the rest of the mice followed a repeating BAAB order. Following the eight training sessions, mice were given a single test session using the same procedure as during training, except that all mice received 4% sucrose solution in each of the two contexts during the test session. The order of presentation of contexts during the test session was fully counterbalanced with respect to the counterbalancing used in training.

#### Licking analysis

In addition to the number of licks, the mean lick cluster size was recorded. A lick cluster was defined as a series of two or more licks made with less than 0.5 s between each lick (i.e., the end of one lick and the start of the next). This criterion is similar to that used by Davis and Smith (1992) in rats.

### Results

For analyses in Experiment 1, and in all subsequent experiments, where the assumption of sphericity was violated a Greenhouse-Giesser correction was used.

#### Training

The mean total licks, lick cluster size and consumption across sessions during the training phase are shown in Table 2. Mice made more licks of the 32% solution in Context B than the 4% solution in Context A, *F*(1, 7) = 26.88, *p* = .001, η_p_^2^ = .79, 95% CI [.24, .89], and similarly consumed more of the 32% solution than the 4% solution, *F*(1, 7) = 79.68, *p* < .001, η_p_^2^ = .92, 95% CI [.58, .96]. Two mice failed to make a cluster of licks in one or more sessions during training and were therefore removed from the analysis of lick cluster sizes. Lick cluster sizes were larger for the 32% solution than the 4% solution, *F*(1, 5) = 12.27, *p* = .017, η_p_^2^ = .71, 95% CI [.01, .85], power = .79.[Table-anchor tbl2]

#### Test: Total licks

**Figure 1 fig1:**
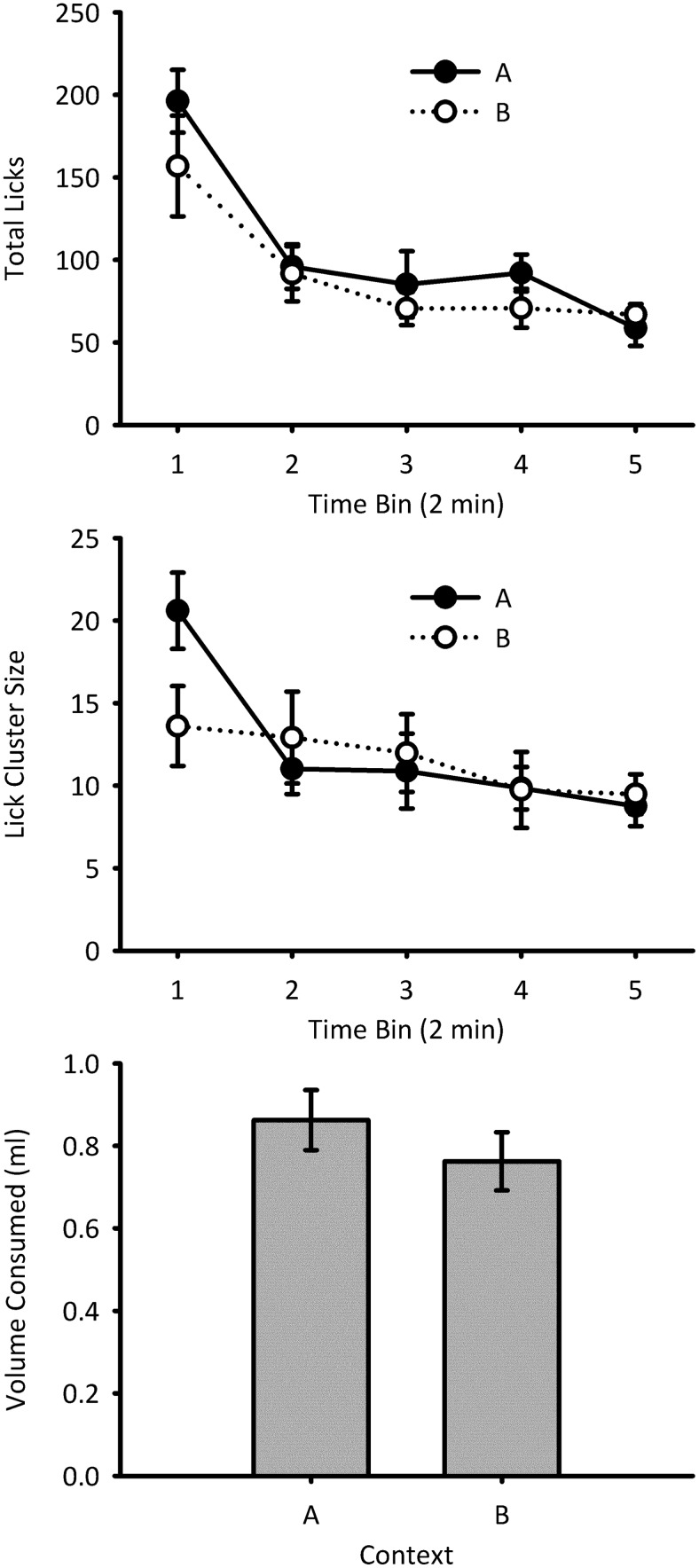
Test data for Experiment 1. Total number of licks (top) and mean lick cluster size (center) are shown in 2-min time bins for each condition. The amount of sucrose solution consumed in each of the two conditions during the test trial is shown in the bottom panel. Error bars indicate ± standard error of the mean.

The total number of licks during the test session in Contexts A and B is shown in Figure 1 (top panel). Licking decreased over time bins, and the number of licks was similar in both contexts. A repeated measures ANOVA of Bin × Context showed a significant main effect of bin, *F*(4, 28) = 19.38, *p* < .001, η_p_^2^ = .73, 95% CI [.48, .80], power = 1.00, but no significant main effect of context, *F*(1, 7) = 1.09, *p* = .33, η_p_^2^ = .13, 95% CI [.00, .51], power = .11, and no significant interaction between bin and context, *F* < 1, power = .15.[Fig-anchor fig1]

#### Test: Lick cluster size

The lick cluster size during consumption of 4% sucrose in Contexts A and B is shown in Figure 1 (center panel). Lick cluster sizes were larger in Context A than B in the first time bin, but were similar in the two contexts thereafter. A repeated measures ANOVA of Bin × Context showed a significant main effect of bin, *F*(4, 28) = 6.55, *p* = .001, η_p_^2^ = .48, 95% CI [.13, .61], power = .98, but no significant main effect of context, *F* < 1, power = .04. However, there was a significant interaction between bin and context, *F*(4, 28) = 3.49, *p* = .020, η_p_^2^ = .33, 95% CI [.01, .49], power = .78. Simple main effects analysis of the interaction showed that lick cluster size was greater in Context A than Context B during the first 2-min bin, *F*(1, 7) = 6.79, *p* = .035, but not for any other bins (*F*s < 1). The lick cluster size changed over the course of the test in Context A, *F*(4, 28) = 10.37, *p* < .001, but this was not the case for Context B, *F*(4, 28) = 1.29, *p* = .30.

#### Test: Consumption

The amount of 4% sucrose consumed in Contexts A and B during the test session is shown in Figure 1 (bottom panel). Consumption was similar in the two contexts, *F*(1, 7) = 1.00, *p* = .35, η_p_^2^ = .13, 95% CI [.00, .50], power = .10.

### Discussion

In accordance with the study by Daniel et al. (2008), it was found that successive negative contrast is context dependent. The shift to the 4% sucrose solution in Context B (previously paired with 32% sucrose) resulted in a negative contrast effect compared with consumption of 4% sucrose in Context A (previously paired with 4% sucrose). However, rather than being an overall reduction in consumption, there was a transient reduction in the mean lick cluster size in the first 2-min bin. This pattern of results replicates our previous findings (Austen & Sanderson, 2015) suggesting that negative contrast primarily affects palatability, as measured by lick cluster size.

## Experiment 2

Whereas exposure to a high sucrose concentration can have a detrimental effect on subsequent consumption of a low sucrose concentration, pairing a high sucrose concentration with a flavor can lead to greater consumption of that flavor, compared with a control flavor, in the absence of the high sucrose concentration (e.g., Dwyer, 2005; Sclafani, Marambaud, & Ackroff, 2014). These results, compared with those of Experiment 1, suggest that flavors and contexts, although both are cues for sucrose, have qualitatively different effects on behavior. However, it is possible that the different effects are not dependent on the nature of the cues but on differences in the training procedures employed with flavor preference conditioning and successive negative contrast. The aim of Experiment 2 was to verify that flavors have a facilitatory, rather than detrimental, effect on consumption using a procedure that was identical to that used in Experiment 1, except that flavors replaced the roles of contexts (see Table 1). Therefore, mice received exposure to a 4% sucrose concentration mixed with Flavor X and exposure to a 32% sucrose concentration mixed with Flavor Y. At test, mice were presented with the 4% solution with Flavor X and Flavor Y. Using a similar procedure to Experiment 1, if flavors have a facilitatory effect, then, at test, mice will show greater consumption of Y than X.

### Method

#### Subjects

Eight female C57BL/6J/Ola mice (Charles River UK Ltd.) were used. Mice were approximately 5 months old at the beginning of the experiment and weighed between 21.8 and 25.6 g (*M* = 23.2 g). The mice had previously been used in an unrelated appetitive, magazine approach, conditioning experiment, conducted in a different room in operant boxes that were distinct from those used in the current experiment. Mice remained on a restricted diet for the duration of testing to maintain their weights at 85% of their initial free-feeding weights. All other housing and husbandry details were identical to those in Experiment 1.

#### Apparatus

The apparatus was identical to that used in Experiment 1, with the exception that there were now eight identical operant chambers. In addition, the sucrose solutions were flavored with either cherry or grape Kool Aid (0.05% wt/vol, Kraft Foods, Inc., Rye Brook, NY).

#### Procedure

The procedure followed the same as for Experiment 1, except that the sucrose concentrations were paired with distinct flavors rather than the distinct auditory and visual contexts used in Experiment 1. Each trial was conducted in the absence of the house light or the clicker, therefore replicating the dark, quiet context in Experiment 1. The allocation of flavors and order of presentations during training and at test were counterbalanced in the same manner as for the contexts in Experiment 1.

### Results

#### Training

Mean total licks, lick cluster size, and consumption across sessions during the training phase are shown in Table 2. Mice made more licks for the 32% solution paired with Flavor Y than the 4% solution paired with Flavor X, *F*(1, 7) = 240, *p* < .001, η_p_^2^ = .97, 95% CI [.83, .98], power = 1.00, and similarly consumed more of the 32% solution than the 4% solution, *F*(1, 7) = 156, *p* < .001, η_p_^2^ = .96, 95% CI [.75, .98], power = 1.00. Two mice failed to make a cluster of licks in one or more sessions during training and were therefore removed from the analysis of lick cluster sizes. Lick cluster sizes were larger for the 32% solution than the 4% solution *F*(1, 5) = 30.5, *p* = .003, η_p_^2^ = .86, 95% CI [.20, .93], power = .99.

#### Test: Total licks

**Figure 2 fig2:**
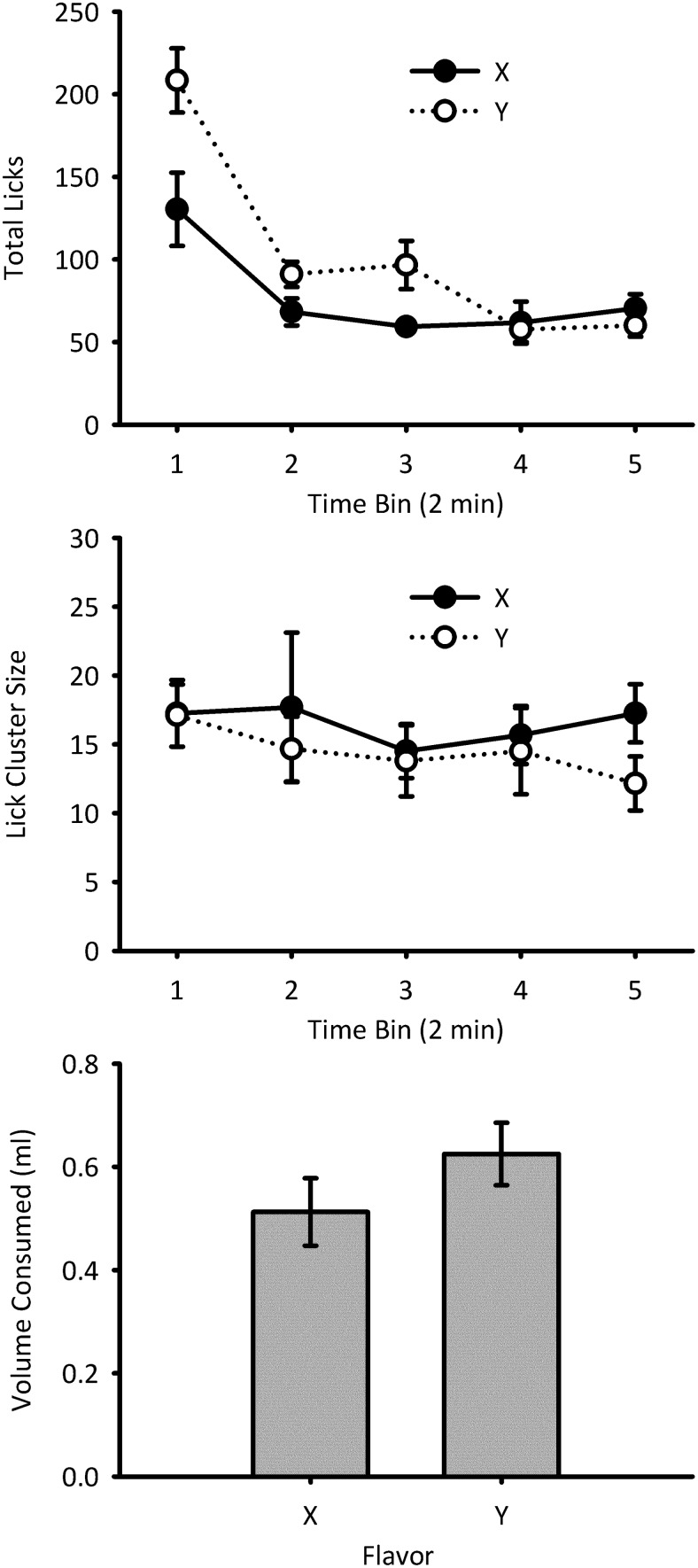
Test data for Experiment 2. Total number of licks (top) and mean lick cluster size (center) are shown in 2-min time bins for each flavor. The amount of sucrose solution consumed in each of the two flavors during the test trial is shown in the bottom panel. Error bars indicate ± standard error of the mean.

The total number of licks of 4% sucrose during the test session for Flavors X and Y is shown in Figure 2 (top panel). Licking declined over the course of the test for both flavors, but during the first three 2-min bins, mice made more licks for Flavor Y than X. A repeated measures ANOVA of Bin × Flavor showed significant main effects of bin, *F*(4, 28) = 35.3, *p* < .001, η_p_^2^ = .83, 95% CI [.66, .88], power = 1.00, and flavor, *F*(1, 7) = 15.3, *p* = .006, η_p_^2^ = .69, 95% CI [.11, .83], power = .91. There was also a significant interaction between these two main effects, *F*(4, 28) = 6.05, *p* = .009, η_p_^2^ = .46, 95% CI [.11, .60], power = .97. Simple main effects analysis of the interaction showed that the total number of licks was higher for Flavor Y than Flavor X during Bins 1, *F*(1, 7) = 10.53, *p* = .014, 2, *F*(1, 7) = 10.32, *p* = .015, and 3, *F*(1, 7) = 8.52, *p* = .022, but not for Bins 4 or 5 (*F*s < 1). The number of licks changed over the course of the test for Flavor X, *F*(4, 28) = 7.16, *p* = .012, and for Flavor Y, *F*(4, 28) = 39.2, *p* < .001.[Fig-anchor fig2]

#### Test: Lick cluster size

The mean lick cluster size during consumption of 4% sucrose in Flavors X and Y during the test session is shown in Figure 2 (center panel). Data from two mice have been removed from the analysis because of being unable to calculate lick cluster size on one or more bins. Mice made similar sized lick clusters for X and Y. A repeated measures ANOVA of Bin × Flavor did not show a significant main effect of bin, *F* < 1, power = .16, or flavor, *F* < 1, power = .06. The interaction between bin and flavor was also not significant, *F*(4, 20) = 1.12, *p* = .38, η_p_^2^ = .18, 95% CI [.00, .34], power = .24. Given the findings of Experiment 1, it may be expected that an effect on lick cluster size would be most likely in the first 2-min bin of the test session. In order to directly assess whether this was the case, a paired-samples *t* test was conducted on the data from the seven out of eight animals for which there were data for bin 1. There was no significant difference in lick cluster size between Flavors X and Y, *t*(6) = 0.07, *p* = .95.

#### Test: Consumption

The volume of 4% sucrose in Flavors X and Y consumed during the test session is shown in Figure 2 (bottom panel). Mice consumed more of Flavor Y than X, *F*(1, 7) = 10.3, *p* = .015, η_p_^2^ = .60, 95% CI [.03, .78], power = .77.

### Discussion

Pairing a flavor with 32% sucrose resulted in greater consumption of that flavor, compared with another flavor paired with 4% sucrose, when both were presented in 4% sucrose at test. These findings replicate other studies in mice showing a flavor conditioning effect (e.g., Sclafani et al., 2014). The results of the test phase suggest that prior exposure to a flavor paired with 32% sucrose led to a conditioned preference for that flavor over the flavor previously paired with 4% sucrose. However, it should also be noted that a consequence of exposure training was that mice consumed a greater amount of the flavor paired with 32% sucrose than the flavor paired with 4% sucrose. It is possible that the greater exposure to the CS+ flavor reduced neophobia to that flavor such that it was preferred at test. However, we have carried out a number of studies (Austen & Sanderson, 2015) examining neophobia to the flavors that were used in the present experiment, using similar exposure training procedures, and have failed to demonstrate a preference in consumption for a familiar over a novel flavor. Therefore, it is unlikely that habituation of neophobia was responsible for the preference conditioning effect.

There was no significant difference in mean lick cluster size between the CS+ and CS– flavors. This result fails to replicate those found in rats showing that CS+ flavors may also elicit a greater number of licks per cluster (e.g., Dwyer et al., 2009), suggesting that, in rats, flavor preference conditioning enhances palatability of the CS+. The failure to find an effect on mean lick cluster size occurred despite the significant difference in the levels of consumption of the CS+ and CS–. These results, compared with those in Experiment 1, provide a double dissociation between the measures of consumption and mean lick cluster size. Context cues (Experiment 1) affected mean lick cluster size but not measures of consumption. Flavor cues affected consumption (overall intake and total number of licks) but not mean lick cluster size.

## Experiment 3

Experiments 1 and 2 show that contexts and flavors have dissociable effects at two levels. First, whereas contexts had a detrimental effect, flavors had a facilitatory effect. Second, context affected lick cluster size but had no effect on levels of consumption (as measured by total licks and overall consumption). However, flavors affected consumption but had no effect on lick cluster size. Although contexts and flavors had seemingly opposing effects, they affected different aspects of licking behavior in a dissociable manner. This may suggest that negative contrast and flavor conditioning are independent, unrelated effects, possibly relating to association formation with different aspects of sucrose, such as its sensory and affective properties. However, it has been claimed that some failures to detect flavor conditioning have been because of negative contrast effects (E. Capaldi et al., 1989). Furthermore, Lucas and Timberlake (1992) demonstrated that contexts and flavors, when combined, have an antagonistic effect on behavioral control. Thus, when context and flavor cues both predicted subsequent availability of a high sucrose concentration, there was no increase in consumption of the flavor, but when only flavor cues were predictive, there was an increase in consumption. These results suggest that contextual cues elicit an anticipatory negative contrast effect that opposes the flavor conditioning effect that animals would otherwise show.

The purpose of Experiment 3 was to test whether flavor and context cues have opposing effects on consumption behavior, or whether they affect independent forms of behavioral expression in a noninteracting manner. During exposure, a similar design to Experiments 1 and 2 was used, but now mice were exposed to a 4% sucrose concentration paired with Flavor X that was always presented in Context A, and a 32% sucrose concentration that was paired with Flavor Y that was always presented in Context B (see Table 1). At test, all mice were presented with the 4% solution in both contexts, but for half of the mice (Group Congruent), the flavor–context pairings were the same as in exposure training (AX and BY), and for the remaining mice (Group Incongruent), the pairings were reversed (AY and BX). If contexts and flavors have opposing effects on consumption, then Group Congruent will show a smaller difference in the measures of consumption between the two context–flavor pairings than Group Incongruent.

### Method

#### Subjects

The experiment was conducted using two cohorts of animals. Both cohorts consisted of 16 female C57BL/6J/Ola mice (Charles River UK Ltd.); however, one mouse from one cohort was not tested because of blindness, and another mouse from the other cohort died during training (the data acquired from this mouse were removed from data analyses), and this had the result that there were 15 mice per between group condition. In the first cohort, mice were approximately 10 weeks of age at the beginning of the experiment and weighed between 15.4 and 20.2 g (*M* = 18.3 g). In the second cohort, mice were approximately five months old at the beginning of the experiment and weighed between 20.6 and 23.9 g (*M* = 21.8 g). This second cohort had previously been used in an unrelated appetitive, magazine approach, conditioning experiment, conducted in a different room in operant boxes that were distinct from those used in the current experiment. Mice were placed on a restricted diet during the course of testing to maintain their weights at 85% of their initial free-feeding weights. All other housing and husbandry details were identical to those in Experiments 1 and 2.

#### Apparatus

The apparatus was identical to that used in Experiment 2.

#### Procedure

Mice were divided into two groups (Group Congruent and Group Incongruent; *N* = 15 per group). During training, all mice received trials in which 4% sucrose was presented in Context A with Flavor X, and trials in which 32% sucrose was paired with Context B and Flavor Y. For approximately half of the animals within each group, Context A was the quiet, dark context, and Context B was the context created by the presentation of the house light and clicker. For the remaining mice, the opposite was true. Within each of these subgroups, for approximately half of the mice, Flavor X was cherry and Flavor Y was grape. The reverse was true for the remaining mice. For approximately half of the mice within these new subgroups, the order of trials during training was a double alternating sequence of AX-BY-BY-AX. For the remaining mice, the sequence was BY-AX-AX-BY. During the test session, all animals received 4% sucrose, with Group Congruent experiencing the same context–flavor contingencies as during training (i.e., AX and BY) and Group Incongruent receiving incongruent contingencies (i.e., AY and BX). The order of the test trials was counterbalanced, as far as possible given the numbers of mice within each condition, with respect to the stimulus allocations and trial orders used in training. All other procedural details were the same as for Experiments 1 and 2.

### Results

#### Training

The mean total licks, lick cluster size, and consumption across sessions during the training phase are shown in Table 2. Mice made more licks for the 32% solution paired with Flavor Y in Context B than the 4% solution paired with Flavor X in Context A, *F*(1, 29) = 145, *p* < .001, η_p_^2^ = .83, 95% CI [.69, .89], power = 1.00, and similarly consumed more of the 32% solution than the 4% solution, *F*(1, 29) = 139, *p* < .001, η_p_^2^ = .83, 95% CI [.68, .88], power = 1.00. Seven mice failed to make a cluster of licks in one or more sessions during training and were therefore removed from the analysis of lick cluster sizes. Lick cluster sizes were larger for the 32% solution than the 4% solution, *F*(1, 22) = 72.1, *p* < .001, η_p_^2^ = .77, 95% CI [.54, .85], power = 1.00.

#### Test: Total licks

**Figure 3 fig3:**
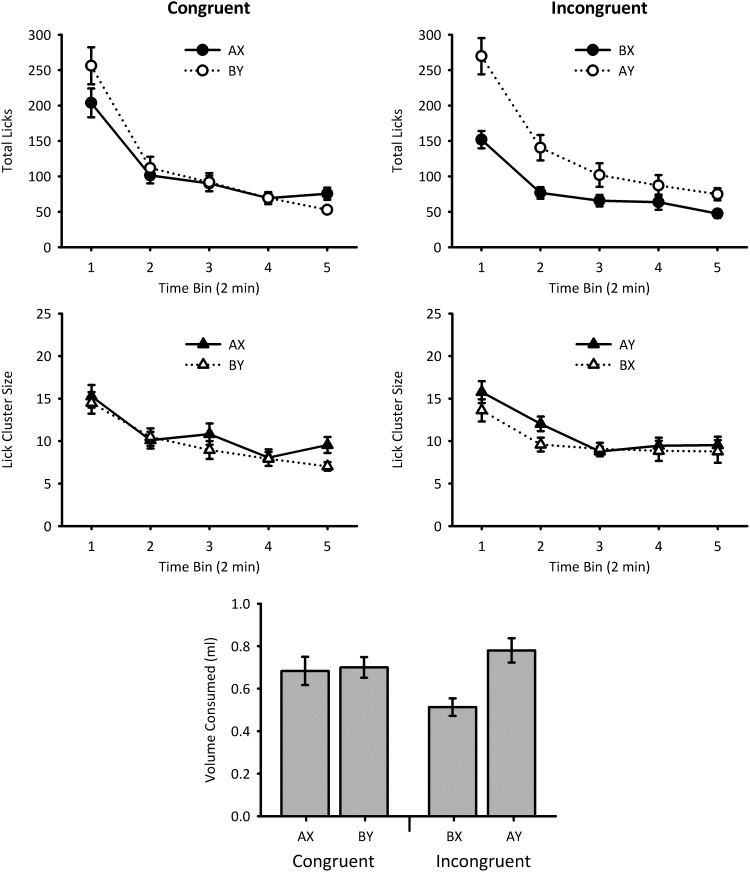
Test data for Experiment 3. Top panel: Total number of licks for Group Congruent (left side) and Group Incongruent (right side). Licking for Flavors X and Y are shown by black and white circles, respectively. Middle panel: Mean lick cluster size for Group Congruent (left side) and Group Incongruent (right side). Mean lick cluster size in Contexts A and B are shown by black and white triangles, respectively. The amount of sucrose solution consumed during AX and BY trials for Group Congruent, and AY and BX trials for Group Incongruent, is shown in the bottom panel. Error bars indicate ± standard error of the mean. Note that for the different measures there is change in the appropriate between group comparisons. For the analyses of total licks and consumption, the responses for AX and BX, and BY and AY, were compared between groups, but for lick cluster size AX and AY, and BY and BX, were compared between groups. Therefore, for total licks and consumption the factor of congruency refers to whether the flavor was presented in a congruent context, whereas for lick cluster size congruency refers to whether the context was paired with a congruent flavor.

The total number of licks of 4% sucrose during the test sessions is shown in Figure 3 (top panels). Licking in both groups declined over the test, but whereas Group Incongruent showed greater consumption on AY than BX trials, Group Congruent showed little difference between BY and AX trials. Given that the number of licks was affected by flavor–sucrose pairings in Experiment 2 and not context–sucrose pairings in Experiment 1, the results were analyzed by conducting a Flavor (X vs. Y) × Context Congruency (Group Congruent vs. Group Incongruent) × Bin ANOVA. Therefore, the variables were nested in such a manner than that the number of licks of Flavors X and Y were directly compared between the two groups as well as within the groups. There were significant main effects of flavor, *F*(1, 28) = 10.2, *p* = .003, η_p_^2^ = .27, 95% CI [.03, .48], power = .82, and bin, *F*(4, 112) = 176, *p* < .001, η_p_^2^ = .86, 95% CI [.81, .89], power = 1.00, and significant interactions between flavor and context congruency, *F*(1, 28) = 5.44, *p* = .027, η_p_^2^ = .16, 95% CI [.00, .39], power = .53, and between flavor and bin, *F*(4, 112) = 8.89, *p* < .001, η_p_^2^ = .24, 95% CI [.10, .34], power = 1.00. The three-way interaction was not significant, *F* < 1, power = .12. Further analysis of the critical Flavor × Context Congruency interaction showed that, for Group Congruent, there was no significant difference between AX and BY, *F* < 1, but there was a difference between BX and AY for Group Incongruent, *F*(1, 28) = 15.3, *p* = .001. Group Congruent made more licks for AX than Group Incongruent made for BX, *F*(1, 28) = 5.57, *p* = .025, but the comparison between BY and AY was nonsignificant, *F*(1, 28) = 1.07, *p* = .31.[Fig-anchor fig3]

#### Test: Lick cluster size

The mean lick cluster sizes made during consumption of 4% sucrose in the test sessions are shown in Figure 3 (center panels). On the whole, lick cluster sizes were very similar across the conditions, but mice made slightly larger lick clusters in Context A than Context B, and this effect was marginally greater in Group Incongruent than Group Congruent. Given that mean lick cluster size was affected by context–sucrose pairings in Experiment 1 and not flavor–sucrose pairings in Experiment 2, the results were analyzed by conducting a Context (A vs. B) × Flavor Congruency (Group Congruent vs. Group Incongruent) × Bin ANOVA. Therefore, the variables were nested in such a manner that the size of lick clusters made in Contexts A and B were directly compared between the two groups as well as within the groups. There was a significant main effect of bin, *F*(4, 112) = 38.1, *p* < .001, η_p_^2^ = .58, 95% CI [.44, .65], power = 1.00. The main effect of context failed to reach significance, *F*(1, 28) = 3.52, *p* = .071, η_p_^2^ = .11, 95% CI [.00, .33], power = .35. All other main effects and interactions—crucially, those involving flavor congruency—were nonsignificant, *F*s < 1.7, *p*s > .17.

#### Test: Consumption

The volume of 4% sucrose consumed during the test sessions is shown in Figure 3 (bottom panel). Group Incongruent consumed more of Flavor Y than Flavor X, but this was not true for Group Congruent. Given that the consumption was affected by flavor–sucrose pairings in Experiment 2 and not context–sucrose pairings in Experiment 1, the results were analyzed by conducting a Flavor (X vs. Y) × Context Congruency (Group Congruent vs. Group Incongruent) ANOVA. Therefore, the variables were nested in such a manner than that the consumption of Flavors X and Y were directly compared between the two groups as well as within the groups. There was a significant main effect of flavor, *F*(1, 28) = 6.71, *p* = .015, η_p_^2^ = .19, 95% CI [.01, .42], power = .63, but no significant main effect of context congruency, *F* < 1, power = .07. However, there was a significant interaction between these two main effects, *F*(1, 28) = 8.62, *p* = .007, η_p_^2^ = .24, 95% CI [.02, .46], power = .75. Simple main effects analysis of the interaction showed that there was a difference in amount of sucrose consumed for Group Incongruent, *F*(1, 28) = 15.3, *p* = .001, but that this was not the case for Group Congruent, *F* < 1. In addition, Group Congruent consumed more of Flavor X than Group Incongruent, *F*(1, 28) = 8.59, *p* = .007, but this was not true for Flavor Y, *F*(1, 28) = 1.21, *p* = .28.

### Discussion

Mice tested in the congruent condition, in which both the context and flavor at test predicted the same sucrose concentration, failed to show a flavor preference conditioning effect, making a similar number of licks and consuming similar amounts in the AX and BY test trials. However, mice tested in the incongruent condition, in which the context–flavor pairings at test (AY and BX) predicted different levels of sucrose concentration, showed greater consumption of Flavor Y, which was previously paired with 32% sucrose, than Flavor X, which was previously paired with 4% sucrose.

There was no significant effect of congruency at test on mean lick cluster size. Therefore, the effect of congruency was limited to the measures of overall consumption. However, the effect of context on mean lick cluster size in the first 2-min bin of the test phase was very small compared with that found in Experiment 1, and the difference was numerically greater in the incongruent group than in the congruent group. Therefore, by comparing the results of the current experiment with those in Experiment 1, it could be speculated that the congruent presentation of flavors and contexts at test did diminish the negative contrast effect on mean lick cluster size, but there was no significant beneficial effect of incongruent context–flavor pairings at test. It is possible that an effect of congruency was not found because novel context–flavor pairings may result in a generalization decrement that affects particular consumption measures (e.g., mean lick cluster size but not total licks), reducing the ability to detect their combined effect on associative retrieval of particular sucrose concentrations.

The results demonstrate that context and flavor cues interact to determine overall levels of consumption. This interaction occurred despite contexts and flavor cues affecting dissociable aspects of consumption behavior when tested separately. In Experiment 1, contexts, in the absence of any flavors, affected mean lick cluster size. In Experiment 2, flavors, when exposed in the same context, affected overall measures of consumption. The interaction between the contexts and flavor cues suggests that, at some level, flavor preference conditioning and successive negative contrast rely on the same processes that, under certain conditions, can be placed in competition with one another.

## General Discussion

The results demonstrate negative contrast and preference conditioning effects in mice, using comparable exposure and test procedures with context and flavor cues, respectively. Whereas negative contrast was demonstrated by a reduction in mean lick cluster size in the absence of any change in overall levels of consumption, flavor preference conditioning resulted in an increase in consumption (as measured by total licks and amount consumed), independent of any significant change in mean lick cluster size. Therefore, these procedures in mice resulted in a double dissociation between the two measures. This is in contrast to results with rats in which changes in both measures may occur (Dwyer et al., 2009; Grigson, Spector, & Norgren, 1994). However, it is often possible to dissociate the two measures within flavor preference conditioning and negative contrast procedures. For example, in flavor preference conditioning in rats, the effect on total licks is more resistant to extinction than the effect on lick cluster size (Dwyer et al., 2009). In contrast, in food-deprived rats, the successive negative contrast effect on total licks extinguishes faster than the effect on lick cluster size (Grigson et al., 1993, 1994). The double dissociation between the two measures in the present study suggests that, at the very least, flavor preference conditioning has a stronger effect on measures of overall consumption than on palatability, whereas the opposite is true for successive negative contrast.

The effects of contexts and flavors on behavior likely reflect differences in the nature of the association formed with sucrose. There is evidence that flavors can enter into associations with multiple components of the properties of sucrose. For example, a flavor–taste and a flavor–calorie association may be formed (E. D. Capaldi, Owens, & Palmer, 1994; Fedorchak & Bolles, 1987; Harris, Gorissen, Bailey, & Westbrook, 2000). Furthermore, the flavor may enter into an association with the affective, hedonic value of sucrose, resulting in an increase in the palatability of the flavor (Forestell & LoLordo, 2003). In the present experiment, the dissociation between consumption and mean lick cluster (a measure of palatability) suggests that flavors formed associations with the taste or potentially calorific nature of sucrose, but they did not acquire the hedonic, palatable properties of sucrose. Contexts, however, did affect the measure of palatability without affecting consumption. The fact that contexts had a negative rather than a positive effect on palatability may suggest that rather than eliciting a conditioned response they controlled habituation of lick cluster size. Thus, contexts may determine conditioned diminution of the unconditioned response (Kimmel, 1966; Wagner, 1976).

Despite the dissociation between contexts and flavors in terms of their effect on specific measures, it was found that there was an interaction between the two types of cue in determining the effect on overall levels of consumption. When CS+ and CS– flavors were trained in separate contexts and then tested in those contexts (AX and BY), mice failed to show an increase in consumption of the CS+ flavor. However, when the flavor–context combinations were switched such that the CS+ flavor was tested in the context previously paired with 4% sucrose, and the CS– flavor was tested in the context previously paired with 32% sucrose (AY and BX), mice did consume more of the CS+ flavor. These results demonstrate that when contexts and flavors are placed in opposition at test (as for Group Congruent), it results in failure to behaviorally express flavor preference learning. A study by Lucas and Timberlake (1992) showed that contexts and flavors can interact with one another in a sequential flavor–sucrose pairing procedure. However, it was not clear from their study whether the interaction occurred in learning (e.g., contexts overshadow flavor–sucrose learning) or whether the interaction resulted in a performance effect. In Experiment 3 of the present study, the difference between the congruent and incongruent conditions in the test phase demonstrates that, given that the two conditions had identical exposure training, the interaction was a performance failure that could be reversed by presenting flavor–context combinations that were incongruent with those used in training.

The interaction between the cues may happen at a number of levels. One possibility is that the context and flavor cues lead to response competition, such that the reduction of lick cluster size in a context previously paired with 32% sucrose reduces the chances of consuming more of that flavor. Indeed, for a mouse to be able to demonstrate both effects (i.e., a reduction in lick cluster size and an increase in total licks), the overall number of clusters would have to increase to overcome the reduction in licks caused by fewer licks per cluster. However, this pattern of effects is certainly possible. It is well established that lick cluster size increases monotonically as a function of sucrose concentration, whereas the number of licks follows an inverted U-shaped function (Davis & Smith, 1992; Spector et al., 1998). Therefore, animals will consume more of an intermediate concentration of sucrose than a high concentration despite the fact that consumption occurs in smaller lick clusters than for the high concentration. As mentioned in the introduction, we have replicated this effect in mice using the same apparatus as the current study (Austen & Sanderson, 2015). Although we cannot rule out a response competition account of the results of Experiment 3, such an account appears unlikely because of the dissociable effects of sucrose concentration on overall levels of consumption and lick cluster size.

A different explanation is that the interaction occurred at the level of memory retrieval, such that the context cue reduced the ability of the flavor cue to retrieve the memory of the unconditioned stimulus (US) that results in preference conditioning. A possible account of this competition may be derived from Wagner and Brandon’s (1989) AESOP model. The model proposes that a CS can enter into an association with separate representations of the sensory and affective properties of a US to different extents, dependent on the temporal arrangement of the cues. For example, the representation of the sensory properties of cues decays faster than the representation of the affective properties. It may be assumed that, as a result of the close temporal correlation between the experience of the flavor and sucrose, flavors may more readily enter into an association with the sensory properties than the affective properties of sucrose. In contrast, contexts may more readily enter into an association with the longer lasting affective properties of sucrose. The model proposes that the nodes that represent the different sensory and affective properties of the US will decay into a secondary activity state, A2, at different rates after the presentation of the US. Presentation of stimuli while the nodes of other stimuli are in the A2 state will result in the formation of inhibitory associations. Given that the context will likely be processed while the sensory node of the US is in the A2 state as a result of recent consumption (e.g., in the intervals between lick clusters), it is possible that, more than just failing to enter into an association with the sensory properties, the context may actually become a conditioned inhibitor of the sensory properties of the US. The result of this is that the context will reduce the ability of the flavor to associatively retrieve the sensory properties of the sucrose, and, consequently, there will be a reduction in conditioned responding. A similar account has been used to explain the excitatory and inhibitory effects of the same CS on different response measures (McNish, Betts, Brandon, & Wagner, 1997).

This analysis suggests that the reason why contexts and flavors produce opposing results is because of the difference in the temporal nature of how they are experienced. Context cues have greater opportunity to form associations with the longer lasting affective properties of sucrose, whereas flavors form associations with the shorter lasting sensory properties. Therefore, it is possible that if cues that form an animal’s context were experienced only when sucrose was consumed, thereby increasing the temporal correlation with the shorter lasting properties of sucrose, then those cues would function like flavor cues and produce preference conditioning effects. Similarly, if the temporal correlation between flavors and sucrose was reduced such that flavors were experienced in between clusters of licks, then flavors may come to act more like contexts and produce negative contrast. To our knowledge, a test of this hypothesis is yet to be done, but it would be of theoretical significance in determining whether the crucial difference between contexts and flavor cues is simply experience of their temporal properties or whether, instead, their difference reflects a preparedness for learning effect (e.g., Garcia & Koelling, 1966) that results in an innate disposition towards contexts more readily producing contrast effects and flavors producing preference effects.

In addition to the present results, Albertella, Harris, and Boakes (2008) have also demonstrated that expression of flavor preference conditioning can be controlled by contexts. In their study, rats showed a preference for almond over water in a context in which they had been exposed to almond, but failed to show this preference when tested in a context in which they had been exposed to sucrose. Albertella et al. (2008) interpreted these results in terms of context-dependent adaptation to sucrose. Thus, rats failed to show a preference in a context in which they had previously been exposed to sucrose because they had adapted to a level of sweetness that rendered them insensitive to the difference between the conditioned properties of almond versus water. The present results may offer an alternative explanation in terms of context-dependent contrast effects. Therefore, rats may have failed to show a preference because of the contrast between the expected concentration of sucrose and the conditioned flavor. Although our results may offer a slightly different interpretation of the findings, it is also possible that context-dependent adaptation is one of the potential mechanisms for negative contrast effects (see Flaherty, 1996, for a discussion).

Although the precise mechanisms of how context and flavor cues interact are yet to be determined, the results demonstrate that consummatory behavior involves a number of potentially competing processes. Importantly, the results provide an insight into the circumstances in which external cues determine changes in eating behavior.

## Figures and Tables

**Table 1 tbl1:** Design of Experiments 1–3

Experiment	Exposure	Test
Experiment 1	A – 4%, B – 32%	A – 4%, B – 4%
Experiment 2	X – 4%, Y – 32%	X – 4%, Y – 4%
Experiment 3		
Congruent	AX – 4%, BY – 32%	AX – 4%, BY – 4%
Incongruent	AX – 4%, BY – 32%	AY – 4%, BX – 4%
*Note.* A and B represent distinct contexts, and X and Y represent distinct flavors. The percentage denotes the concentration of sucrose with which the cues were paired during exposure and test.		

**Table 2 tbl2:** Mean (Standard Error of the Mean) Total Licks, Lick Cluster Size, and Consumption Across Sessions for Each Condition in the Training Phase of Experiments 1–3

			Lick	
Experiment	Condition	Total licks	cluster size	Consumption (ml)
Experiment 1	A	242 (30)	10.4 (.9)	.37 (.05)
	B	599 (68)	18.0 (2.1)	.84 (.04)
Experiment 2	X	246 (9)	11.6 (.7)	.32 (.02)
	Y	771 (37)	21.7 (1.5)	.91 (.06)
Experiment 3	AB	259 (18)	9.6 (.5)	.36 (.02)
	XY	664 (37)	19.5 (1.2)	.75 (.04)
*Note.* A and B represent distinct contexts, and X and Y represent distinct flavors.
