# Correction to Austen and Sanderson (2016)

**DOI:** 10.1037/xan0000105

**Published:** 2016-07

In the article “Contexts Control Negative Contrast and Restrict the Expression of Flavor Preference Conditioning,” by Joseph M. Austen and David J. Sanderson (*Journal of Experimental Psychology: Animal Learning and Cognition,* 2016, Vol. 42, No. 1, pp. 95–105. http://dx.doi.org/10.1037/xan0000091), in Table 2 the conditions in Experiment 3 are labeled AB and XY, but they should be AX and BY.

